# Individual differences in oscillatory brain activity in response to varying attentional demands during a word recall and oculomotor dual task

**DOI:** 10.3389/fnhum.2015.00381

**Published:** 2015-06-29

**Authors:** Gusang Kwon, Sanghyun Lim, Min-Young Kim, Hyukchan Kwon, Yong-Ho Lee, Kiwoong Kim, Eun-Ju Lee, Minah Suh

**Affiliations:** ^1^Center for Neuroscience Imaging Research (CNIR), Institute for Basic Science (IBS), Sungkyunkwan UniversitySuwon, South Korea; ^2^Department of Health Sciences and Technology, Samsung Advanced Institute for Health Science and Technology (SAIHST), Sungkyunkwan UniversitySeoul, South Korea; ^3^Center for Biosignals, Korea Research Institute of Standards and ScienceDaejeon, South Korea; ^4^Department of Medical Physics, University of Science and TechnologyDaejeon, South Korea; ^5^School of Business, Sungkyunkwan UniversitySeoul, South Korea; ^6^Department of Biomedical Engineering, Sungkyunkwan UniversitySuwon, South Korea; ^7^Department of Biological Science, Sungkyunkwan UniversitySuwon, South Korea

**Keywords:** working memory capacity, dual-task, oculomotor task, word recall task, MEG and EEG, alpha band desynchronization, frontal midline theta

## Abstract

Every day, we face situations that involve multi-tasking. How our brain utilizes cortical resources during multi-tasking is one of many interesting research topics. In this study, we tested whether a dual-task can be differentiated in the neural and behavioral responses of healthy subjects with varying degree of working memory capacity (WMC). We combined word recall and oculomotor tasks because they incorporate common neural networks including the fronto-parietal (FP) network. Three different types of oculomotor tasks (eye fixation, Fix-EM; predictive and random smooth pursuit eye movement, P-SPEM and R-SPEM) were combined with two memory load levels (low-load: five words, high-load: 10 words) for a word recall task. Each of those dual-task combinations was supposed to create varying cognitive loads on the FP network. We hypothesize that each dual-task requires different cognitive strategies for allocating the brain’s limited cortical resources and affects brain oscillation of the FP network. In addition, we hypothesized that groups with different WMC will show differential neural and behavioral responses. We measured oscillatory brain activity with simultaneous MEG and EEG recordings and behavioral performance by word recall. Prominent frontal midline (FM) theta (4–6 Hz) synchronization emerged in the EEG of the high-WMC group experiencing R-SPEM with high-load conditions during the early phase of the word maintenance period. Conversely, significant parietal upper alpha (10–12 Hz) desynchronization was observed in the EEG and MEG of the low-WMC group experiencing P-SPEM under high-load conditions during the same period. Different brain oscillatory patterns seem to depend on each individual’s WMC and varying attentional demands from different dual-task combinations. These findings suggest that specific brain oscillations may reflect different strategies for allocating cortical resources during combined word recall and oculomotor dual-tasks.

## Introduction

Attention and working memory capacity (WMC) are closely related because focused attention ensures stable encoding of external stimuli, a critical step for successful WMC performance. In this respect, attention is a very critical element of various cognitive processes because it allows our brains to allocate limited cortical resources when faced with varying task demands. Brain oscillations have been well studied in terms of their function and relation to attention and WMC (Niebur et al., 1993; Başar et al., 1999; Klimesch, 1999; Herrmann and Knight, 2001; Sauseng et al., 2010; Benchenane et al., 2011; Roux and Uhlhaas, 2014). In this study, we attempted to measure individual differences in behavioral performance and neural activation using a dual-task paradigm combining oculomotor and word recall tasks while using simultaneous MEG and EEG recordings. Among the different subtypes of brain waves, we focused on theta (4–6 Hz) and alpha (8–12 Hz) rhythms in this study because of their well-known association with both WMC and attention-related functions (Klimesch et al., 1997b; Stam, 2000; Aftanas and Golocheikine, 2001; Sauseng et al., 2006).

Alpha band activity reflects various attentional processes (Ray and Cole, 1985; Sauseng et al., 2005; Gould et al., 2011; Klimesch, 2012; Belyusar et al., 2013), and many studies report alpha band desynchronization or attenuation in relation to the attentional demands of the task conditions (Gevins et al., 1998; Klimesch et al., 2006; Higashima et al., 2007; Maclean and Arnell, 2011). In general, increased task or attentional demands are known to be associated with decreases in posterior alpha power (Gevins et al., 1997; Krause et al., 2000; Stipacek et al., 2003); in particular, the upper alpha band (10–12 Hz) has been used to measure more detailed differences in memory load or task complexity (Krause et al., 2000; Doppelmayr et al., 2005; Jaušovec and Jaušovec, 2012). The upper alpha band is the most sensitive to semantic memory processing demands or task-specific effects (Klimesch, 1999, 2000; Klimesch et al., 2006). Explanations of this phenomenon assume that attenuation of alpha power reflects a release of inhibition related to complex activation processes (Klimesch et al., 2007; Klimesch, 2012) and enhanced information transformation in the thalamo-cortical circuits, which reflect upper alpha oscillations in the retrieval processes of (semantic) long-term memory (Klimesch, 1999; Stipacek et al., 2003).

Theta band activity has been well investigated for its implications in memory performance (Raghavachari et al., 2001; Fuentemilla et al., 2010). In particular, a major role of theta activity in WMC function has been consistently reported (Tesche and Karhu, 2000; Raghavachari et al., 2006; Sauseng et al., 2010). During the encoding and retention period, theta activity shows a strong increase with memory load, and these WMC load-dependent theta activities occur in the frontal area (Missonnier et al., 2006; Maurer et al., 2015). Therefore, frontal theta activity has been investigated as an index of WMC load (Jensen and Tesche, 2002; Onton et al., 2005; Itthipuripat et al., 2013; Hsieh and Ranganath, 2014). Thus, alpha power decreases in the posterior site, and frontal theta power increases represent the general index of EEG (or MEG) with increasing cognitive demands in various tasks requiring attentional demands or memory processes.

Many studies have investigated individual differences in alpha band desynchronization created by the interactions between WMC, intelligence, and cortical activation (Grabner et al., 2004; Doppelmayr et al., 2005; Neubauer et al., 2006; Caravaglios et al., 2015). However, conflicting results exist concerning the way this cortical activation is manifested between individuals with different intelligence. Some studies show that highly intelligent subjects exhibit more alpha event-related desynchronization (ERD) and that the larger ERD is associated with good performance (Klimesch et al., 1997a; Jaušovec and Jaušovec, 2004; Doppelmayr et al., 2005). The opposite has also been reported: more intelligent subjects showed smaller alpha ERD at posterior sites (Neubauer et al., 1995, 1999; Grabner et al., 2006). According to the neural efficiency hypothesis, these individuals are more efficient in inhibiting task-irrelevant brain areas (Klimesch et al., 2006). Additionally, this hypothesis asserts that efficiency is derived from the disuse of task-irrelevant brain areas and the more focused use of task-relevant areas. Frontal midline (FM) theta band activity (henceforth, FM-theta) was also reported as an index for measuring individual differences (Gevins and Smith, 2000; Zakrzewska and Brzezicka, 2014). In those studies, high-ability subjects showed an enhanced FM-theta EEG signal during high WM load conditions, which suggests that the subject can better focus and sustain attention on the task than a low-ability subject. Zakrzewska and Brzezicka, 2014) explain the FM-theta as an individual trait that can reflect an individual WM mechanism, i.e., neural efficiency.

In our dual-task paradigm, we combined a word recall task of varying cognitive load (5 or 10 words) with an oculomotor task composed of two types (predictive and random) of a smooth pursuit eye movement (P-SPEM and R-SPEM) task and an eye fixation (Fix-EM) task. We hypothesized that varying levels of attentional demand would be generated from each dual-task combination. The neural networks modulating SPEM are known to overlap somewhat with those relevant to WM and attention, such as the frontal eye fields (FEF), the supplementary eye fields (SEF) in the frontal areas, the prefrontal cortex (PFC) and the parietal cortex, including the intraparietal sulcus (IPS) and superior parietal lobule (SPL), the cerebellar areas and the MT complex (medial temporal area (MT), and the middle superior temporal area (MST; Culham et al., 1998; Chen et al., 2002; Lencer et al., 2004; Barnes, 2008; Makin et al., 2012). High cognitive function, such as attention, can affect the outcome of SPEM (Van Donkelaar and Drew, 2002; Hashimoto et al., 2004; Hutton and Tegally, 2005; Madelain et al., 2005) and the activation of SPEM also affects cognitive outcomes (Schütz et al., 2007, 2008; Lovejoy et al., 2009; Lee et al., 2011). Therefore, we hypothesized that word recall and an oculomotor dual-task would activate a common fronto-parietal (FP) network as well as the cortico-cerebellar network.

Lee et al. (2011) found that P-SPEM improves word recall performance in dual-task situations, and they suggest that this phenomenon arises from the synergic activation of shared neural networks. The degree of cognitive influence on the oculomotor outcome can be affected by the behavioral and neural strategies of individuals possessing different WMCs. We pursued this avenue of investigation further by exploring whether different oculomotor tasks and cognitive loads under dual-task situations can specifically influence brain oscillations in the frontal and parietal areas. Furthermore, we aimed to compare behavioral performance between high- and low-WMC groups and identify the underlying neural mechanisms during the dual-task.

## Materials and Methods

### Participants

Seventeen right-handed university students (age: 23.4 ± 2.7 years old; eight males) participated in this study in return for a monetary incentive. They were all informed of the experimental procedure, and we collected written consent and a questionnaire concerning their physical condition before the experiment. This study was approved by the university ethics committee (Sungkyunkwan University, Suwon, South Korea).

#### Neuropsychological Assessment

Before the main MEG and EEG sessions, a total number of 33 participants were screened with regard to their WMC by administering a well-established test: the Korean version of the California Verbal Learning Test (K-CVLT). This test is well known as a validated neuropsychological tool for assessing verbal learning and WM (Kim and Kang, 1999). Based on standardized test scores, we selected eight individuals whose scores were within the 0–25th percentile (the low-WMC group), and nine individuals whose scores were over the 75th percentile (the high-WMC group). We excluded the remaining 16 participants with intermediate scores from the main experiment.

#### Behavioral Data Analysis

Word recall was used to assess behavioral performance. Trial results in which the number of recalled words were less than one word in the low-load (five words) condition or two words in the high-load (10 words) condition were excluded from further analysis as outliers. The proportion of outlier trials for high- and low-WMC groups was not significantly different (high: 14.8% and low: 16.7%, *p* = 0.66, two-tailed).

### Electrophysiological Recording

We simultaneously recorded MEG and EEG during the dual-task. MEG recording was conducted with a 152-channel first-order axial gradiometer MEG system (Korea Research Institute of Standards and Science, Daejeon, South Korea), and EEG was recorded with a 32-channel EEG system (Biosemi, Netherlands). The EEG electrodes were placed according to the international 10/20 system and the electrode offset was kept below 20 mV. Additionally, we attached two EOG channels, a vertical one below the left eye and a horizontal one lateral to the right eye to monitor eye movement and blinking. MEG and EEG signals were acquired with a pass-band filter from 0.01–100 Hz with a 60 Hz notch filter and DC to 400 Hz and sampled at 512 Hz. The whole experiment was conducted in a magnetically and electrically shielded room. We performed the coil location calibration to compensate for any possible head movement during the break.

### Dual-task Design

**Figure 1:**
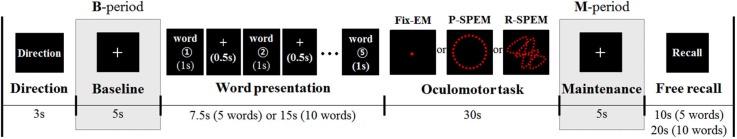
**The diagram for the dual-task paradigm**. Three different types of oculomotor tasks were preceded by word presentation (5 or 10 words). Analysis of MEG and EEG data was focused on the maintenance period (M-period), normalized by the baseline period (B-period).

Each participant had a total of six separate sets of dual-tasks. Each dual task set consisted of a verbal WM task, which required remembering 5 or 10 Korean words followed by one of the three types of oculomotor tasks lasting for 30 s. Oculomotor tasks included Fix-EM and both predictive and random smooth P-SPEM and R-SPEM, respectively. Figure 1 illustrates the dual-task paradigm.

The criteria for the Korean word selection were usage frequency and ease of comprehension (Cho, 2003). We defined five words as the “low-load” condition and 10 words as the “high-load” condition. We created a total of 18 word sets: nine sets for low-load and another nine sets for high-load. Every word set was constructed homogeneously in terms of the number of syllables and the level of difficulty. Each participant was tested with the same 18-word sets. Each set was presented and combined with one of three oculomotor tasks in random fashion.

The detailed procedures for each oculomotor task can be found in the work of Lee and colleagues (Lee et al., 2011). In short, the R-SPEM task involved the eye tracking of a freely moving target with unpredictable trajectories, whereas the P-SPEM involved eye tracking of a moving target alongside 12 predictable, circular trajectories with a speed of 0.4 Hz. Target speed was maintained at a constant 10 °/s. Fix-EM involved eye fixation on a central red dot. Each oculomotor task took approximately 30 s to complete, and we asked all subjects to conduct each oculomotor task carefully.

The words were visually presented on a screen with a central fixation cross between adjacent words. The screen was located 50 cm ahead of the subject. During the oculomotor tasks and the following maintenance period, the subjects were required to remember the words and then recall as many of them as possible (regardless of the presented order) during the recall period. We manually wrote down the words they correctly recalled after the experiment by listening to voice recordings.

We temporally separated the encoding, maintenance and recall periods in our dual-task paradigm. During each oculomotor task, the subjects were allowed to blink, and their eye movements were monitored through CCD camera installed in a shielded room to ensure their oculomotor behavior was well controlled. Each experimental set was composed of six trials, which were presented in a randomized order for each subject; each trial consisted of either a low- or high-load word recall condition combined with one of the three oculomotor tasks. Each set lasted for 6.5 min and was repeated three times per subject. The subjects were allowed to take a break between the sets.

### Data Analysis

Both MEG and EEG data were analyzed using the Fieldtrip toolbox developed at the Donders Institute for Brain, Cognition, and Behavior (Oostenveld et al., 2011), as well as custom made scripts (Matlab 7, Mathworks). The EEG data were re-referenced to the Cz channel for further analysis. Data analysis was performed only during the baseline (B-period) and maintenance (M-period) periods. Artifacts including eye blinking and heart beat were removed using Independent component analysis (ICA). Both MEG, and EEG data were bandpass filtered (twopass Butterworth filter) at 1–70 and 0.1–30 Hz, respectively.

#### Spectral Analysis

We conducted time-frequency analysis based on a sliding time window (steps of 10 ms) for the data segments of the B- and M-periods. We applied an adaptive time window containing four cycles for each frequency (ΔT = 4/f), resulting in an adaptive spectral smoothing of Δf = 1/ΔT, using a single Hanning taper. This procedure was applied equally for MEG and EEG data. For the MEG analysis, a planar gradient was calculated to simplify the interpretation of the sensor-level data, in which the signal amplitude is largest directly above the source. The resulting power values for the horizontal and vertical components of the planar gradient were combined. The power estimates of each M-period were normalized using the power estimate of the B period from the same trial and the averaged power estimates were compared among each condition. We restricted our data analysis to the theta (4–6 Hz) and upper alpha (10–12 Hz) frequency bands.

#### MEG Data Realignment

Because the MEG sensor data are not aligned across each trial, i.e., the location of the head relative to the MEG sensors are different across subjects, we transformed each MEG data towards a standard gradiometer location using the “ft_megrealign” function in the Fieldtrip. The standard gradiometer was determined using the average of all gradiometer information of each subject. This transformation makes it easier to compare MEG data across subjects.

#### Statistical Analysis

For the EEG data, we averaged the absolute power spectral estimates of the target frequency band in a single electrode, i.e., theta band (4–6 Hz) in Fz and upper alpha band (10–12 Hz) in Pz during the M-period. We used PASW statistics 18 (SPSS Inc., Chicago, IL, USA) for statistical analysis.

For the MEG data, we compared the oscillatory power of different conditions using a nonparametric cluster-based permutation test (Maris and Oostenveld, 2007). By using a Monte-Carlo randomization method, this test controls for type-I error occurring from multiple comparisons by clustering neighboring sensors that show the same effect over time, space, and frequency. The maximum of the cluster-level summed *t*-values was used as the test statistic for a randomization null-distribution. This distribution was approximated and repeated 500 times using the observed data. An accurate estimate of the Monte Carlo *p* value was obtained using 500 random draws, and statistically significant differences were calculated when the *p*-value was smaller than the critical alpha level of 0.05 (two-sided test).

## Results

### Behavioral Results

**Figure 2:**
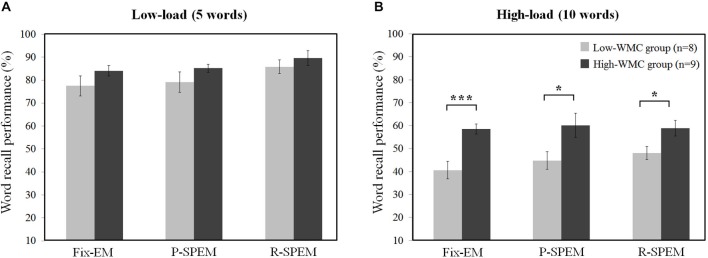
**Individual differences of word recall performance (%) over oculomotor tasks in low-load (A) and high-load (B) condition**. *p < 0.05, ***p < 0.001.

We converted each word recall result into the percent performance for statistical comparison between low- and high-load conditions. We tried to investigate the effects of the oculomotor task on word recall performance. Furthermore, we subdivided the results of the word recall performances into high- and low-WMC groups in each memory load condition to see how their WMC affects the influence of each oculomotor task on word recall performances. For a 2 × 3 × 2 repeated measures ANOVA with the between-subjects factor *group* (high- and low-WMC) and with two within-subject factors—the *oculomotor task* (Fix-EM, P-SPEM and R-SPEM) and *load* (high- and low-load)—we found no significant three-way interaction (*F*_(2,30)_ = 0.125, *p* = 0.883). However, we found statistically significant main effects of the *oculomotor task* (*F*_(2,30)_ = 5.263, *p* = 0.011), the *load* (*F*_(1,15)_ = 307.813, *p* < 0.001) and the *group* (*F*_(1,15)_ = 8.709, *p* = 0.01). For the oculomotor task effect in particular, word recall performance in the R-SPEM condition was significantly higher than that in the Fix-EM condition (Bonferroni adjusted *p* = 0.007). The interaction between the *group* × *load* factor was also significant (*F*_(1,15)_ = 6.507, *p* = 0.022), and these results show that the group differences depend on the memory load, i.e., the high-WMC group showed better word recall performances than the low-WMC group only in the high-load condition. Figures 2A,B represents the word recall performances of high- and low-WMC groups under six different dual-task combinations. The group difference in each oculomotor condition was all significant under high-load conditions: Fix-EM (*t*_(15)_ = −5.633, *p* < 0.001), P-SPEM (*t*_(10)_ = −2.796, *p* = 0.019), R-SPEM (*t*_(15)_ = −2.291, *p* = 0.037), whereas in the low-load condition no statistically significant differences were found using *post hoc* independent sample students’ *t*-tests. The original results of the correctly recalled number of words in each condition are given in Table 1.

**Table 1:** **The number of correctly recalled words in each dual-task scenario (mean ± SEM)**.

	Oculomotor task condition	Fix-EM	P-SPEM	R-SPEM
Low-load (5 words)	All (*n* = 17)	4.12 ± 0.14	4.21 ± 0.13	4.37 ± 0.11
	High-WMC (*n* = 9)	4.26 ± 0.19	4.26 ± 0.2	4.48 ± 0.15
	Low-WMC (*n* = 8)	3.9 ± 0.2	4.1 ± 0.18	4.25 ± 0.16
High-load (10 words)	All (*n* = 17)	5.07 ± 0.22	5.49 ± 0.26	5.58 ± 0.19
	High-WMC (*n* = 9)	5.83 ± 0.29	6.13 ± 0.38	5.96 ± 0.24
	Low-WMC (*n* = 8)	4.15 ± 0.23	4.55 ± 0.21	4.95 ± 0.28

### EEG Results

#### Oculomotor Task, WMC, and Oscillatory Power

**Figure 3:**
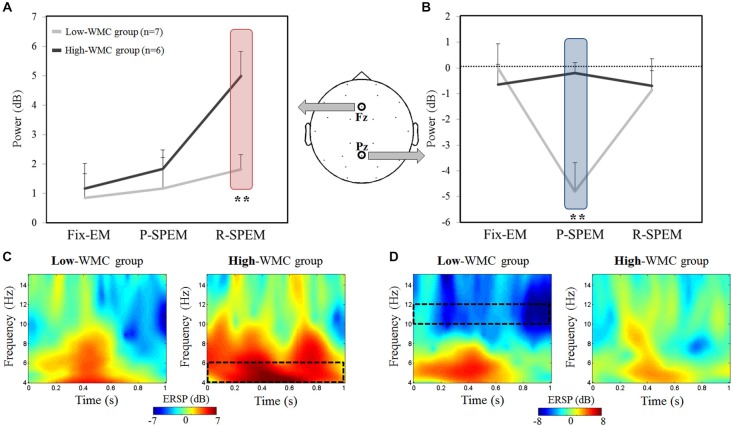
**The averaged EEG theta power (4–6 Hz) at the Fz channel (A) and the averaged upper alpha power (10–12 Hz) at the Pz channel (B) during high-load condition for each oculomotor task in the high- and low-working memory capacity (WMC) groups**. The power was averaged for the first second of the word maintenance period. Time-frequency representations showing the significant differences between the high- and low-WMC groups [red rectangle from **(A)** and blue rectangle from **(B)**] in the R-SPEM condition at the Fz channel **(C)** and the P-SPEM condition at the Pz channel **(D)**. The spectrotemporal window used for power averaging is represented with a black dotted rectangle.

We compared the oscillatory EEG power during the M-period (normalized by the B-period) between the high- and low-WMC groups in each dual-task condition. We found that noticeable oscillatory activities of the theta (4–6 Hz) and upper alpha (10–12 Hz) bands existed during the early phase of the M-period. Therefore, we focused on the 0–1 s time interval of the M-period. Figure 3 illustrates the averaged oscillatory power at the Fz and Pz in the high-load condition, as well as frequency representations showing significant differences between the high- and low-WMC groups. We used a 2 × 3 × 2 repeated measures ANOVA with the between-subjects factor *group* (high- and low-WMC) with two within-subject factors—*oculomotor task* (Fix-EM, P-SPEM and R-SPEM) and *load* (high- and low-load)—for statistical analysis. For a theta band power, the interaction effect between the *oculomotor task* and the *group* was marginally significant (*F*_(2,22)_ = 3.157, *p* = 0.062) and the main effect was significant for the *oculomotor task* (*F*_(2,22)_ = 8.314, *p* = 0.002). This interaction indicates that the differential effect of the oculomotor task is dependent on the WMC group. For the oculomotor task effect, in particular, theta power in the R-SPEM was significantly higher than the Fix-EM and marginally higher than the P-SPEM condition (Bonferroni adjusted *p* = 0.005 and 0.066, respectively). The group difference in each oculomotor task condition was only significant in the R-SPEM condition (*t*_(11)_ = −3.38, *p* = 0.006) using *post hoc* independent sample Student’s t-tests (Figure 3A). These patterns were only observed in the high-load condition but not in the low-load condition, which is supported by significant three-way interaction (*F*_(2,22)_ = 3.912, *p* = 0.035). For an alpha band power using same statistical procedures as we did with theta band, the interaction between the *oculomotor task* and the *group* was statistically significant (*F*_(2,22)_ = 9.181, *p* = 0.002) and the main effect was significant for the *oculomotor task* (*F*_(2,22)_ = 6.075, *p* = 0.012). More specifically, alpha power in the P-SPEM was marginally higher than the Fix-EM condition (Bonferroni adjusted *p* = 0.053). The group difference was only significant in the P-SPEM condition (*t*_(11)_ = −3.64, *p* = 0.004; Figure 3B). Three-way interaction was also significant (*F*_(2,22)_ = 5.297, *p* = 0.013), which means, again, that these patterns were found only in the high-load condition. Overall, frequency distribution of frontal and parietal EEG oscillation for the two groups confirms our statistical results (Figures 3C,D).

#### Correlation Between Neuropsychological Score

**Figure 4:**
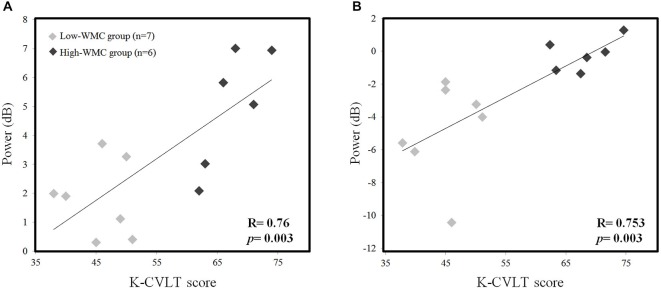
**Correlation between the K-CVLT score and averaged EEG theta power (4–6 Hz) at the Fz channel (A), and the averaged EEG upper alpha power (10–12 Hz) at the Pz channel **(B)** in high-load conditions during the word maintenance period (0–1 s)**.

We examined the correlation between the K-CVLT score and the absolute theta, upper alpha power during the early phase of the M-period (0–1 s) in order to find the relationship between WMC and oscillatory power. We found a positive correlation in both Fz theta power (*r* = 0.76, *p* = 0.003) and Pz upper alpha power (*r* = 0.753, *p* = 0.003) with the K-CVLT score (Figure 4), and this significant correlation was not observed in the low-load scenario.

### MEG Results

**Figure 5:**
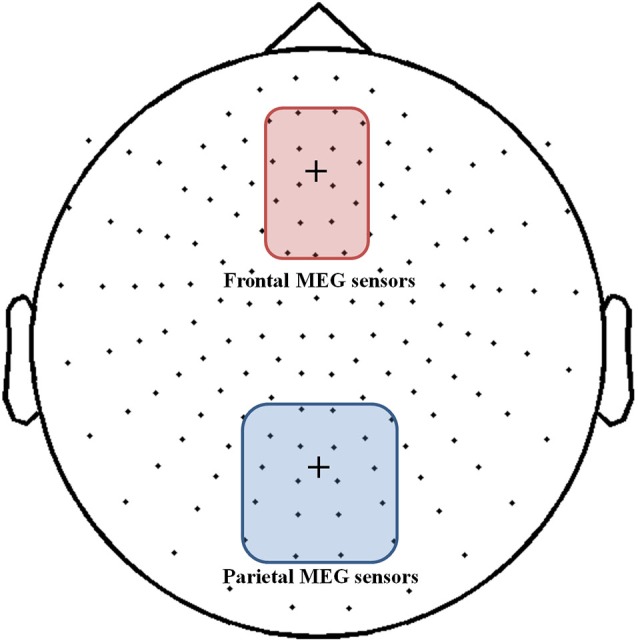
**MEG 152 sensor montage with the 17 frontal (red round rectangle) and 18 parietal (blue round rectangle) sensors represented**. The fixation cross in each rectangle indicates the location of the EEG Fz and Pz electrodes.

**Figure 6:**
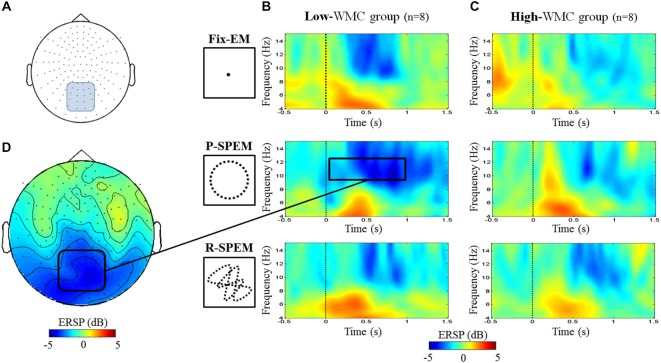
**MEG 152 sensor montage representing the parietal sensors (blue round rectangle) (A) and the time-frequency representations for the averaged power of parietal sensors for each oculomotor condition of the low- (B) and high- (C) WMC groups during high-load condition**. The upper alpha band desynchronization of the P-SPEM condition in the low-WMC group (10–12 Hz, 0–1 s, black rectangle) was distributed over parietal regions **(D)**.

As we transformed MEG sensor data into a standard gradiometer location, we classified frontal and parietal MEG sensors according to their relative location to the EEG Fz and Pz electrodes, respectively. Considering both distance and direction from the corresponding electrode (Fz, Pz), we determined 17 frontal and 18 parietal MEG sensors (Figure 5). We focused on finding global patterns of frontal theta synchronization and parietal upper alpha desynchronization during dual-tasks. From the time-frequency representations, we could observe prominent upper alpha desynchronization for the low-WMC group in the P-SPEM with high-load conditions (Figures 6B,C) within the same spectrotemporal window (0–1 s, 10–12 Hz), we used for the EEG analysis. As an exploratory statistical analysis of the transformed MEG data, we found condition pairs reaching a significance level of *p* < 0.05 with the non-parametric cluster-based permutation test. The significant differences in parietal upper alpha desynchronization were found between the Fix-EM and the P-SPEM in the low-WMC group (850–1500 ms) and between the high- and low-WMC groups in the P-SPEM condition (140–300 ms). There were no significant differences in the frontal theta synchronization under any dual-task conditions.

## Discussion

In this study, we used a word recall and oculomotor dual-task paradigm to investigate the effect of varying levels of attentional demand on neural activity and the behavioral performance of normal subjects. We discovered that behavioral outcomes could be affected by the distinct neural strategy between high- and low-WMC groups during dual-task situations. Using simultaneous MEG and EEG recording, we found prominent desynchronization patterns in the upper alpha band (10–12 Hz) in the parietal area during the P-SPEM condition. This upper alpha power attenuation was shown only in the low-WMC group performing the high-load task. We presuppose that alpha band desynchronization is modulated by the different levels of attentional demand during dual-tasks. In addition, we observed significantly higher FM-theta power in the high-WMC group experiencing the random SPEM with the high-load condition, and these differences were only observed in the EEG results (Figure 3). In combination with the parietal alpha power differences, we argue that individual neural efficiency affects differentiated behavioral results and neural strategies between high- and low-WMC groups during dual-tasks. In addition, different types of oculomotor tasks, as well as WM load, affect the attentional demands and create those differences.

### Different Neural Strategies Generated From Different Oculomotor Tasks

The two different types of SPEM, the P-SPEM and the R-SPEM, might exert different neural strategies during dual-tasks, and our neural data support this hypothesis. Smooth pursuit tracking needs the activation of multiple brain areas on the FP network and cortico-cerebellar tract (such as the FEF, SEF, lateral intraparietal area, and vermis; Tanabe et al., 2002; Krauzlis, 2005; Orban de Xivry and Lefèvre, 2007; Contreras et al., 2011). However, during the P-SPEM, eye movement becomes automatic due to the high predictability of the target trajectory; consequently, it relies more on the cerebellar output because the cerebellum plays a dominant role in generating anticipatory and predictive movement (Ivry, 2000; Nitschke et al., 2005; Thier and Ilg, 2005; Leggio and Molinari, 2015). Therefore, the P-SPEM may evoke different involvement from FP and fronto-cerebellar network than non-predictive smooth pursuit tasks such as random tracking. Accordingly, the P-SPEM may reduce the load imposed on the FP network. Ramnani (2006) reported that during the acquisition of any cognitive tasks that become increasingly automatic, a decrease in prefrontal activity will be accompanied by increasing activity of connected areas, including Crus II in the cerebellum. Hayter et al. (2007) also reported that the interaction between the prefrontal areas and the cerebellar cortex facilitates the execution of routine information processing, thus freeing prefrontal circuitry to prepare for additional tasks. In this regard, the repeated target predictability during the P-SPEM may induce automatic oculomotor control toward the end of the task, which saves cortical resources. Thus, the subjects could devote those spared cortical resources during the word maintenance period, which requires selective attention to maintain the words. The alpha desynchronization of the parietal MEG and EEG data demonstrates this phenomenon. In particular, in the P-SPEM with high-load condition, individuals with a low WMC showed a larger upper alpha band desynchronization in the parietal area during the early phase of the maintenance period than under any other conditions. Moreover, the fact that individuals with low WMC demonstrated higher attenuation than the high-WMC individuals may indicate that the low-WMC group benefits most from “cortical resource saving” with the involvement of the cerebellum.

Conversely, the R-SPEM relies more on the FP network to modify visual errors occurring from the constantly changing target position. In our previous study (Lee et al., 2011), we hypothesized that more extensive cortical resources need to be allocated during the R-SPEM, and the EEG results (Figure 3) demonstrate this by showing the highest FM-theta activity in the high-WMC group during the R-SPEM with high-load conditions than any others. Gevins et al. (1997) found that FM-theta power directly increased with the increase in WM load (i.e., the number of items to be maintained in WM) in both verbal and spatial WM tasks. Therefore, we could conclude that the highest cortical load demands by the complex R-SPEM combined with the high-load in our dual-task design manifested in the increase of FM-theta activity, but only in the high-WMC group.

Finally, the Fix-EM may impose more loads on the FP network than the P-SPEM. The Fix-EM could be perceived as automatic processing like the P-SPEM; however, compared to the P-SPEM, it is a passive viewing process rather than attentive tracking. Therefore, it may not require as much cerebellar involvement because it does not generate as much predictive and anticipatory movement. Our MEG results in the low-WMC group (Figure 6) show that the alpha desynchronization pattern lasts longer in the P-SPEM than in the Fix-EM condition.

Overall, those two eye movements—the Fix-EM and the R-SPEM—may not require the same level of cerebellar involvements as the P-SPEM. The different neural mechanisms of these three eye movements may generate different cortical allocation strategies when combined with a word recall task that shares a common FP network and competes for the limited cortical resources. We could not find significant differences in theta activity in the frontal MEG sensors during the R-SPEM conditions. This may indicate that the theta activity originates from radial dipole because MEG has low sensitivity to radial sources. We need further source-level MEG and EEG analysis in relation to the FM-theta activity.

### Cortical Resource Allocation Depends on WMC

In this study, the high- and low-WMC groups exhibited different behavioral performances and neural activation patterns. The high-WMC group showed better word recall performances than the low-WMC group only in the high-load condition, and the differences were statistically significant over all oculomotor conditions (Figure 2). In our previous study using dual-task paradigm with a seven words recall task (Lee et al., 2011), the difference was only significant in the R-SPEM condition between the high- and low-WMC groups. The number of words may be responsible for the difference in behavioral results in the two studies by affecting the cognitive loads during the dual-tasks. However, we could still verify the role of individual WMC in determining the extent of word recall performance affected by the secondary oculomotor task. The high-WMC group showed superior word recall performance than the low-WMC group regardless of the oculomotor task types in the high-load condition, which requires different levels of cognitive resource demands.

Many clinical papers reported oculomotor disturbances as indicators of a conversion from mild cognitive impairment (MCI) to Alzheimer’s disease (AD; Pereira et al., 2014) and a wide scope of other neurodegenerative disorders (Anderson and MacAskill, 2013; Pinkhardt et al., 2014). Another study demonstrated that mild traumatic brain injury (TBI) patients who are suffering from shearing injuries on the FP network and cortico-cerebellar tract show both oculomotor and cognitive impairment in dual-tasks involving the P-SPEM condition (Suh et al., 2006a,b; Ghajar and Ivry, 2008). In contrast, healthy subjects showed improvement under the same conditions. Therefore, individual differences are an important factor in controlling a dynamic allocation strategy utilizing limited neural resources under dual-task demands. In this study, we also demonstrated this by showing different neural activation between the high- and low-WMC groups. Our EEG results (Figure 3) showed significant differences in Fz theta (4–6 Hz) and Pz upper alpha (10–12 Hz) power during the R-SPEM and the P-SPEM, respectively. Furthermore, the correlation between the K-CVLT score and those two power indices was significant (Figure 4). A recent study showed a linear increase in the FM-theta power in accordance with WM load during maintenance, but only in the high-WMC group (Zakrzewska and Brzezicka, 2014). They explain this phenomenon by associating higher WMC with efficient information processing.

Additionally, the neural efficiency hypothesis focuses on alpha desynchronization in the parietal area in relation to intelligence and cortical activation (Doppelmayr et al., 2002; Grabner et al., 2004; Jaušovec and Jaušovec, 2005). In the MEG results (Figure 6), we could see that under the P-SPEM condition, alpha desynchronization starts earlier in the low-WMC group than in the high-WMC group. Together with the EEG results, these patterns between the high- and low-WMC groups may reflect the different neural strategies that are needed to process the varying levels of attentional demands required in each dual-task condition. Furthermore, neither statistically significant differences in parietal alpha desynchronization nor FM-theta activation between high- and low-WMC groups were found in the low-load condition, which may not require as much attentional demand as the high-load condition.

In this study, we mainly focused on neural responses reflecting WM network involvement under oculomotor network co-activation. However, the effects of WM load on the oculomotor task are also an important aspect in our dual-task paradigm. In our previous paper (Lee et al., 2011) discussing eye tracking behavior in a very similar paradigm with our current study, we have shown that no significant eye velocity errors exist between high- and low-WMC groups for three oculomotor tasks (Fix-EM, P-SPEM and R-SPEM). The velocity error represents how stable the subject’s tracking was during eye movement. However, we found significantly higher velocity errors during the P-SPEM than in the other two oculomotor tasks when the results of high- and low-WMC groups were combined. We interpreted these results to mean that the highly predictable characteristic of the target leads to gaze-leading during the P-SPEM, and this would account for the velocity errors. Additionally, we measured the phase error during the P-SPEM performance, and the results showed the low-WMC group leading the target, whereas the high-WMC group lagged behind the target. These results showed the differential effects of WM on the oculomotor task as well as WMC on the oculomotor behavior.

In conclusion, an individual’s dynamic resource allocation strategy depends on his/her WMC; the individuals with high WMC exerted more efficient processing, which resulted in different behavioral performance and a distinct neural activation pattern compared to the low-WMC individuals during dual-task situations. A recent study by Walshe et al. (2015) also investigated the underlying cognitive and neural processes in younger and older subjects as they performed dual tasks that required different levels of cognitive control. The aim of this study was to find individual differences in brain oscillatory activity during dual-tasks that limited the capacity for cognitive control. The distinct patterns of theta and alpha power over FP areas between the high- and low-WMC groups during the word maintenance period indicated different neural and cortical resource allocation strategies. Furthermore, the findings that neural differences depend on the oculomotor task supports our assumption that distinct neural mechanisms and attentional demands during dual-tasks affect the load imposed on the FP network at different levels. Our results may contribute to the perception that alpha desynchronization in the parietal area and FM-theta synchronization are neural indices for measuring individual differences in response to the varying levels of attentional demands during dual-task situations. Therefore, we suggest that our designed dual-task could have useful applications in the field of cognitive neuroscience and as a diagnostic tool for clinical use.

## Conflict of Interest Statement

The authors declare that the research was conducted in the absence of any commercial or financial relationships that could be construed as a potential conflict of interest.
